# Octreotide treatment of patients with hepatocellular carcinoma - a retrospective single centre controlled study

**DOI:** 10.1186/1756-9966-28-142

**Published:** 2009-11-03

**Authors:** Maximilian Schöniger-Hekele, Joachim Kettenbach, Markus Peck-Radosavljevic, Christian Müller

**Affiliations:** 1 Universitätsklinik für Innere Medizin III, Klinische Abteilung für Gastroenterologie und Hepatologie, Medical University of Vienna, Vienna, Austria; 2 Universitätklinik für Radiodiagnostik, Medical University of Vienna, Vienna, Austria

## Abstract

**Background:**

Studies of treatment with octreotide of patients with hepatocellular carcinoma (HCC) gave conflicting results. We analyzed retrospectively the survival of our patients treated with octreotide monotherapy and compared it to stage-matched patients who received either TACE, multimodal therapy or palliative care.

**Methods:**

95 patients seen at the department of Gastroenterology and Hepatology, Medical University of Vienna with HCC in BCLC stage A or B, who received either TACE, multimodal therapy, long-acting octreotide or palliative care were reviewed for this retrospective study.

**Results:**

Survival rates of patients with BCLC stage B and any "active" treatment (long-acting octreotide, TACE or multimodal therapy) were significantly higher (22.4, 22.0, 35.5 months) compared to patients who received palliative care only (2.9 months). Survival rates of patients with BCLC stage A and "active" treatment (31.4, 37.3, 40.2 months) compared to patients who received only palliative care (15.1 months) did not show statistically significant differences. Octreotide monotherapy showed a similar outcome compared to patients who received TACE or multimodal therapy.

**Conclusion:**

Survival under octreotide treatment was not different compared to TACE or multimodal therapy and might be a therapeutic option for patients with HCC.

## Introduction

Hepatocellular carcinoma is a sequel of chronic liver disease and shows high and increasing prevalence worldwide. In most cases it is associated with the presence of liver cirrhosis and has a poor prognosis with an overall median survival of 8 months in Austria [1]. To assess the survival of patients with hepatocellular carcinoma different prognostic models have been developed [2-5]. Although no staging system has emerged as standard, one of the most widely used survival model is the BCLC (Barcelona Clinic Liver Cancer) staging system [2] which appears to be the most comprehensive, as it links staging to treatment [5]. Treatment options which aim to obtain clinical cure include liver resection, liver transplantation and various forms of local ablation, such as percutaneous ethanol injection (PEI) or radiofrequency ablation. These treatment modalities are only available for patients with early stage hepatocellular carcinomas (patients with BCLC stage A and B). Patients who present with an advanced stage of HCC (Patients with BCLC stage C) will currently be treated, among other modalities, with transarterial chemoembolisation (TACE) or the multi-thyrosin-kinase inhibitor sorafenib [6]. This treatment aims to prolong survival while maintaining the best possible quality of life. Other patients with advanced hepatocellular carcinoma may participate in clinical studies for new treatment modalities or substances, respectively.

One substance which has been discussed controversially in the last years is octreotide. Somatostatin and its synthetic analogues, octreotide and lanreotide are potentially active against HCC due to their antiproliferative and apoptosis-inducing activity; in addition HCC has been shown to overexpress somatostatin receptors on the cell surface [7-10]. Several years ago Kouroumalis et al [11] published a randomized controlled trial which showed a significantly improved survival in patients with inoperable hepatocellular carcinoma treated with octreotide as compared to placebo (13.0 versus 4.0 months). In addition, a second randomized placebo-controlled trial [12] showed an improved survival (49.0 versus 28.0 weeks) and quality of life in patients with advanced hepatocellular carcinoma treated with long-acting octreotide. In contrast, Yuen et al [13] did not find a survival benefit of octreotide-monotherapy in patients with advanced hepatocellular carcinoma. Similarly, a large German study [14] reported an equally poor median survival in the treatment group (4.7 months) and the control group (5.3 months), respectively. It is interesting to note that in the two negative studies [13,14] the median survival of octreotide treated patients and the control group was extremely poor making it difficult to show any possible influence of octreotide treatment on survival. In contrast, in the two positive studies [11,12] survival even in the placebo arms was considerably longer suggesting differences in patient selection.

Due to these divergent study results concerning the influence of octreotide on survival we decided to analyze retrospectively the survival of our patients with hepatocellular carcinoma and octreotide monotherapy and compared it to stage-matched patients who received either TACE, multimodal therapy or palliative care.

## Patients and methods

### Patient characteristics

The charts of all patients with hepatocellular carcinoma (HCC) seen at the department of Gastroenterology and Hepatology, Medical University of Vienna from 1992 to 2004 were reviewed for this retrospective study. At the time of diagnosis 95 of these patients were in BCLC [2] stage A or B and received either TACE, multimodal therapy, long-acting octreotide [Sandostatin LAR] or palliative care. Stage A patients receiving that treatment modalities declined other treatment options such as liver transplantation or resection or were not candidates for this procedures due to older age or concomitant diseases. We focused on reporting the results of stage A and B patients because most of the patients in advanced BCLC stages (stage C and D) received only palliative care and no active treatment at that time. Furthermore, patients with advanced BCLC stages typically suffer from complications of terminal liver cirrhosis which has a considerable influence on survival. To minimize the influence of the underlying liver disease and to focus on the impact of tumour treatment on survival, patients with advanced BCLC stages were excluded. MELD scores within the BCLC stages A and B, respectively and various treatment modalities were not statistically significant when tested allowing for multiple comparisons (p = 0.07).

The demographic data and clinical characteristics are given in Table 1. Liver cirrhosis was diagnosed either by histology or by the typical combination of laboratory tests, clinical and gastroscopic findings and typical signs of liver cirrhosis in CT or ultrasound. Diagnosis of hepatocellular carcinoma was done according to the criteria of EASL [15] and AASLD [16]. Histologic confirmation was performed in 31 of 40 (77.5%) patients in BCLC stage A and 50 of 55 (90.9%) patients with BCLC stage B. Overall, hepatocellular carcinoma was histologically confirmed in 85.3% of our patients.

**Table 1:** Characteristics of patients with HCC according to treatment modalities

		Sandostatin LAR		TACE		multimodal therapy		palliative care	
		
		BCLC A	BCLC B	BCLC A	BCLC B	BCLC A	BCLC B	BCLC A	BCLC B
		
number		11	14	5	9	7	10	17	22
Sex									
	male	6	10	4	8	7	9	14	16
	female	5	4	1	1	0	1	3	6
liver cirrhosis									
	no	0	0	0	2	1	0	2	2
	yes	11	14	5	7	6	10	15	20
Child-Pugh-classification									
	Child A	9	12	1	3	5	8	7	7
	Child B	2	2	4	4	1	2	8	13
	Child C	0	0	0	0	0	0	0	0
MELD (median/range)		7.33 (0.27-15.98)	7.61 (0.16-15.0)	14.60 (11.13-17.35)	11.46 (6.68-16.90)	11.56 (6.78-49.26)	8.98 (7.15-16.01)	12.31 (4.66-58.20)	13.20 (1.53-49.82)
Etiology									
	Alcoholism	5	8	2	4	5	3	8	8
	chronic hepatitis B	1	0	0	0	1	0	2	0
	chronic hepatitis C	4	4	3	2	1	7	5	7
	others	1	2	0	3	0	0	2	6
Age (median/range)		66.5 (53.7-80.5)	68.7 (49.4-73.4)	64.9 (63.6-69.2)	68.4 (48.4-78.4)	50.5 (47.4-64.6)	69.9 (61.2-76.9)	68.5 (43.1-81.2)	62.5 (44.8-73.4)

### Treatment modalities

#### Long-acting Octreotide [Sandostatin LAR]

30 mg long-acting octreotide (Sandostatin-LAR™, Novartis, Basel, Switzerland) was given i.m. once a month until death.

This therapy was given within the context of an unpublished study to compare the clinical outcome of additional percutaneous ethanol instillation (PEI) against no further treatment in patients with HCC, all receiving hormonal treatment with long-acting octreotide. All patients (n = 25) who received only treatment with long-acting octreotide were included in this retrospective comperative study. Patients who received a combination therapy with long-acting octreotide and percutaneous ethanol instillation (PEI) were excluded.

#### Transarterial (Chemo-) embolization (TAE/TACE)

Transarterial (Chemo-) embolization (TAE/TACE) as therapy (n = 17) was chosen in patients with BCLC stage B (advanced tumor without evidence of distant metastases or vessel invasion). Furthermore, patients with BCLC stage A were treated with transarterial embolization (TAE) or transarterial chemoembolization (TACE) in case of contraindications for orthotopic liver transplantation (OLT), liver resection or percutaneous local therapy.

TAE was performed according to a standardized technique. The femoral artery was cannulated under local anesthesia, and diagnostic angiography of the celiac trunk and superior mesenteric artery was performed. After identification of the vascular anatomy, a superselective catheter was pushed forward into the hepatic arteries by use of a guide wire. Afterwards, different mixtures of substances for embolization were used during the time period we analyzed in this retrospective study. First, there was a mixture of N-butyl-2-cyanoacrylate (Histoacryl blue; B. Braun, Melsungen, Germany) and ethiodized oil (Lipiodol Ultrafluide; Guerbet, Villepinte, France) as an embolic agent. Secondly in case of TACE a mixture of doxorubicin and ethiodized oil (Lipiodol Ultrafluide; Guerbet, Villepinte, France) as an embolic agent was used.

TAE/TACE was performed superselectively by occluding only the tumor-feeding segmental arteries or selectively by occluding the right or left hepatic artery. In general, a superselective embolization was aimed. However, in patients with a large tumour mass or more than one nodule in the same lobe, selective embolization of the entire lobe was performed. In patients with tumor disease in both the right and the left liver lobe, only one lobe was embolized during one treatment session to avoid a prolonged postembolization syndrome or postinterventional liver failure. A completion arteriogram was obtained to confirm occlusion of the embolized vessels. After TAE/TACE, the patients were carefully observed and side-effects of embolization were treated symptomatically. Follow-up was done with contrast-enhanced CT of the liver to assess the effect of embolization on the tumor. Depending on success of the already performed interventions embolization sessions were repeated in intervals from 1 to 3 months.

#### Multimodal therapy

Multimodal therapy (n = 17) included a combination of local ablative therapies such as percutaneous ethanol instillation (PEI), radiofrequency ablation therapy or cryotherapy on the one hand and transarterial embolization therapy as described above on the other hand. Usually percutaneous ablative therapies were given first, after signs of tumour progression were seen treatment was continued with TAE/TACE.

#### Palliative care

39 patients received only symptomatic therapy but no active treatment for hepatocellular carcinoma. Patients in this group rejected the offered local ablative therapies, embolization therapy or systemic treatment but opted for palliative care only.

### Statistical analysis

Chi^2 ^test was used to compare proportions and Mann Whitney U tests to compare median values between groups. Survival times were estimated using the Kaplan-Meier method and the differences were tested with the log-rank test. Analysis was performed with Statistica (StatSoft, Inc. (2004). STATISTICA (data analysis software system), version 6. http://www.statsoft.com).

## Results

### Patients with BCLC stage A

40 patients were classified to BCLC stage A. Treatment modalities in this group were: long-acting octreotide [Sandostatin LAR] (n = 11 [27.5%]), TACE (n = 5 [12.5%]), multimodal therapy as defined above (n = 7 [17.5%]) and palliative care only (17 [42.5%]).

### Median Survival (Figure 1)

**Figure 1:**
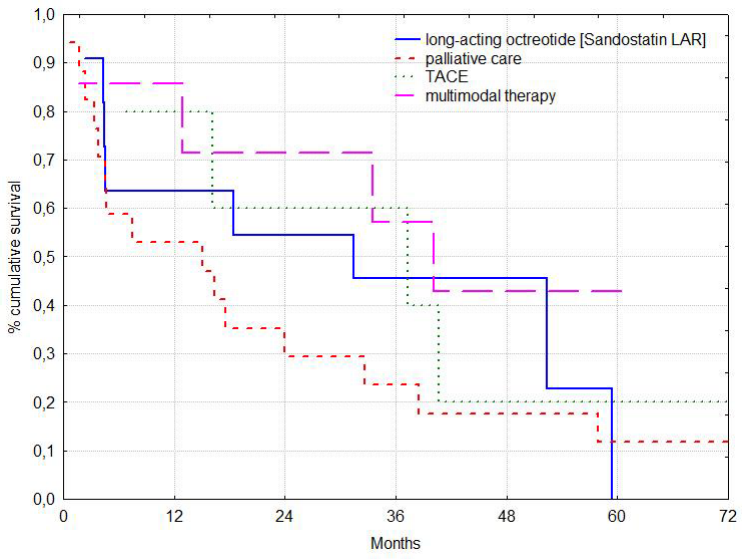
**Patients with hepatocellular carcinoma and BCLC stage A**. Median survival rates in long-acting octreotide [Sandostatin LAR], TACE, multimodal therapy and palliative care were 31.4, 37.3, 40.2 and 15.1 months respectively. Survival rates of patients with active treatment did not differ significantly.

Overall median survival was 18.4 months. Median survival rates in long-acting octreotide [Sandostatin LAR], TACE, multimodal therapy and palliative care were 31.4, 37.3, 40.2 and 15.1 months respectively (Table 2). Although survival rates of patients with "active" treatment (long-acting octreotide [Sandostatin LAR], TACE or multimodal therapy) were more than twice as long as of patients who received only palliative care this difference was not significant. Survival rates of patients with various active treatment modalities did not differ significantly.

**Table 2:** Patient survival according to BCLC stage and treatment

		BCLC A			BCLC B		
		
		number	median survival (months)	log rank test	number	median survival (months)	log rank test
number treatment modalities		40			55		
	Sandostatin LAR	11	31.4	P = 0.35038	14	22.4	P = 0.00003
	TACE	5	37.3		9	22.0	
	multimodal therapy	7	40.2		10	35.5	
	palliative care	17	15.1		22	2.9	

The 1 year survival rate in the long-acting octreotide [Sandostatin LAR] group was 64% and in patients who received multimodal therapy, TACE, and palliative care 86%, 80% and 53%, respectively. The 2 year survival rate in the long-acting octreotide [Sandostatin LAR] group was 55% and in patients who received multimodal therapy, TACE, and palliative care 82%, 60% and 29%, respectively.

### Patients with BCLC Stage B

55 patients were classified as BCLC stage B. These patients received long-acting octreotide [Sandostatin LAR] (n = 14 [25.4%]), TACE (n = 9 [16.4%]), multimodal therapy as defined above (n = 10 [18.2%]) and palliative care (n = 22 [40.0%]), respectively.

### Median Survival (Figure 2)

**Figure 2:**
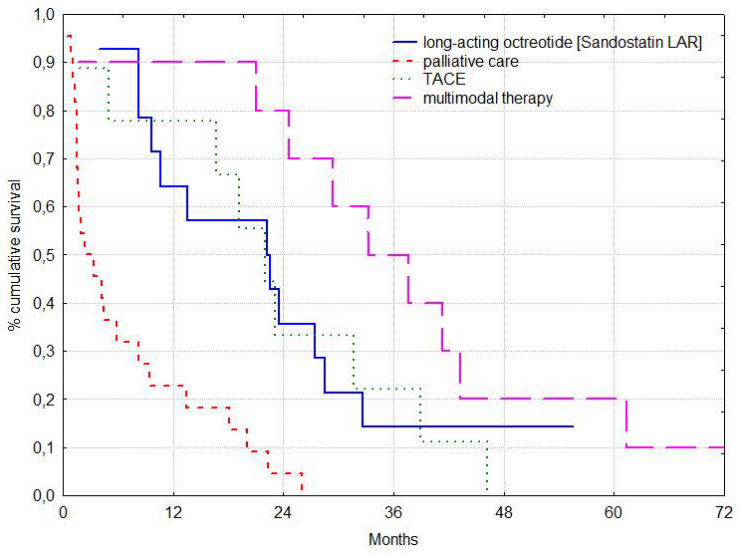
**Patients with hepatocellular carcinoma and BCLC stage B**. Median survival rates in long-acting octreotide [Sandostatin LAR], TACE, multimodal therapy and palliative care were 22.4, 22.0, 35.5 and 2.9 months respectively. Median survival among patients with "active" treatment did not show significant differences (log rank test: P > 0.05).

Overall median survival was 15.1 months. Median survival rates of the group receiving long-acting octreotide [Sandostatin LAR], TACE, multimodal therapy and palliative care were 22.4, 22.0, 35.5 and 2.9 months, respectively (Table 2). Survival rates of patients with "active" treatment (long-acting octreotide [Sandostatin LAR], TACE or multimodal therapy) were significantly higher than of patients who received palliative care only (log rank test: P = 0.00043, P = 0.00151, P = 0.00005). Median survival among patients with various "active" treatment forms did not show significant differences (log rank test: P > 0.05).

The 1 year survival rate in the long-acting octreotide [Sandostatin LAR] group was 64% and in patients who received multimodal therapy, TACE, and palliative care 90%, 78% and 23%, respectively. The 2 year survival rate in the long-acting octreotide [Sandostatin LAR] group was 36% and in patients who received multimodal therapy, TACE, and palliative care 80%, 34% and 5%, respectively.

## Discussion

In the present paper we studied retrospectively the influence of octreotide monotherapy (long-acting octreotide [Sandostatin LAR]) on survival of patients with hepatocellular carcinoma and compared it to BCLC stage-matched patients who received either TACE, multimodal therapy or palliative care only. Our data showed that survival rates of patients with BCLC stage B and any "active" treatment (long-acting octreotide [Sandostatin LAR], TACE or multimodal therapy) were significantly higher as compared to patients who received palliative care only. Although survival time of patients with BCLC stage A and "active" treatment (long-acting octreotide [Sandostatin LAR], TACE or multimodal therapy) were more than twice as long as of patients who received palliative care only this difference was not statistically significant. Median survival among patients with various forms of "active" treatment did not show significant differences (BCLC stage A and B; log rank test: P > 0.05). In particular, octreotide monotherapy showed a similar outcome compared to patients who received TACE or multimodal therapy.

Kouroumalis et al [11] for the first time published a patient population with advanced liver disease (only 3.6% of the patients had Child-Pugh stage A) and HCC treated with octreotide. The treatment group had an excellent median survival of 13.0 months as compared to 4.0 months in the control group, suggesting a beneficial effect of octreotide treatment in this patient population. Similarly, Dimitroulopoulos et al [12] recently reported the results of a randomised placebo-controlled trial which showed a significantly higher survival in somatostatin receptor positive patients receiving long-acting octreotide [Sandostatin LAR] as compared to placebo.

In contrast, the patient population in the randomized controlled trial of Yuen et al [13] had a very poor survival of only 1.93 months in the treatment group and 1.97 months in the control group, respectively. This very poor survival in treatment and control group is remarkable because the majority (51.4%) of the patients included in the treatment group had stage A according to the Child-Pugh classification. Besides, only 8.6% of these patients were in Child-Pugh stage C and 17.1% in Okuda stage III. Therefore the poor outcome of these patients is not reflected in both the Child-Pugh classification (8.6% Child-Pugh Stage C) and the Okuda staging system (17.1% in Okuda stage III). However, nearly half of the patients had a portal vein thrombosis corresponding to advanced disease BCLC stage C and the poor median survival of less than 2 months in treatment and control group indicates terminal liver disease. Finally, due to the bad survival 13 out of 35 patients from the treatment group died before receiving a single dose of long-acting octreotide [Sandostatin LAR]. It is obvious that a positive effect of Sandostatin LAR could only be expected in patients receiving some minimal doses of Sandostatin LAR. Therefore, it seems that the patients in the study of Yuen [13] did not live long enough to benefit from Sandostatin LAR therapy. Similarly, the overall poor survival in both treatment and placebo controlled groups of the recently published HECTOR study (Becker et al [14]) might be the reason for the inability of detecting a survival difference between these two groups. However, also two recent studies could not demonstrate a statistically significant survival benefit in patients with advanced hepatocellular carcinoma treated with long-acting octreotide [Sandostatin LAR] [17,18].

The expression of somatostatin receptors is variable and only 41% of HCC express this receptor on the cell surface [7]. Recently, Bläcker et al [19] showed that in HCC mostly somatostatin receptor subtype III and V are expressed. On the other hand Reyneart found somatostatin receptor I and II expressed on HCC [20]. Given that heterogeneity in expression of somatostatin receptor subtypes both the antiproliferative effect of octreotide and the response rate might be determined by the expression level of various somatostatin receptors on HCC which seems to be independent of histology, underlying liver disease or tumour stage [17]. This might explain differences of the therapeutic effects on survival by long-acting octreotide [Sandostatin LAR] reported in the literature. Indeed Dimitroulopoulos et [12] al showed recently that patients with Somatostatin receptor high expressing tumours survived longer than patients with low expression.

TACE treatment has been shown to improve survival of patients with HCC in a metaanalysis of randomized controlled trials [21,22]. It is surprising that in our retrospective study survival of patients with long-acting octreotide [Sandostatin LAR] alone was similar to TACE treatment or multimodal treatment. Although a selection bias cannot be completely excluded, the patients were comparable as tumour stage, overall liver function and clinical performance status, variables comprising the BCLC staging system, are concerned. The therapeutic potential of octreotide is further stressed by the fact that BCLC stage-matched patients receiving no active treatment had a shorter survival time than patients with TACE treatment as expected from the well known fact of a survival benefit of TACE therapy [19,20]. And yet, TACE treatment was not better than octreotide treatment. Along the same line, the study of Plentz et al [23] showed a similar survival of patients treated with octreotide compared to patients treated with TACE.

Treatment with long-acting octreotide [Sandostatin LAR] was excellently tolerated except for a few episodes of soft stools presumably due to the effect of reduced exocrine pancreatic output. This could easily be corrected either with supplementation of pancreatin containing capsules or with loperamid tablets. No intramuscular haematoma formation was observed after i.m. administration of long-acting octreotide [Sandostatin LAR] despite reduced coagulation capacitiy.

The interpretation of our data might be limited by the retrospective non-randomised nature of our study and the long time period of recruitment of patients which results in a considerable heterogeneity of the study groups. Although, we tried to match the patients in the study groups according to the BCLC system, the best available prognostic staging system, residual heterogeneity in the study population might have influenced the results. In addition, patients under octreotide treatment tended to have lower MELD scores than patients undergoing other treatment modalities although there was no overall difference in MELD score between the various groups.

In summary, this retrospective analysis of survival of BCLC stage-matched patients with HCC showed that octreotide treatment produces a similar survival benefit as TACE or multimodal therapy as compared to no active treatment. Given the few side effects of long-acting octreotide [Sandostatin LAR] this treatment seems to be a therapeutic option for patients with HCC and needs further randomised controlled studies in BCLC stage-matched patients.

## Competing interests

The authors declare that they have no competing interests.

## Authors' contributions

JK performed chemoembolization. MPR recruited patients. MSH and CM were equally involved in the design of the study, patient recruitment, management of the patients, statistical analysis and drafted the manuscript. All authors read an approved the final manuscript.
